# Expediency

**Published:** 1896-07

**Authors:** 


"EXPEDIENCY."—It is a specious plea. Almost any absurdity, a crime even, can be excused, but not *justified*, upon the plea of "expediency." The tramp is hungry; he has no money; he will not work; he must eat. He steals a loaf; it is expedient; there is no other alternative, except starvation. The State Association is "hungry," but we hold there are other alternatives; the emergency does not drive them to commit this *crime against medicine.*

